# Use of orthogonal serine integrases to multiplex plasmid conjugation and integration from *E. coli* into *Streptomyces*

**DOI:** 10.1099/acmi.0.000291

**Published:** 2021-12-08

**Authors:** Hong Gao, Margaret C. M. Smith

**Affiliations:** ^1^​ Department of Biology, University of York, York YO10 5DD, UK; ^2^​ School of Health and Life Sciences, Teesside University, Middlesbrough TS1 3BA, UK; ^3^​ National Horizons Centre, Teesside University, Darlington DL1 1HG, UK

**Keywords:** biosynthesis gene cluster, conjugation, multiplexed integration, natural product, serine integrase, *Streptomyces*

## Abstract

Some major producers of useful bioactive natural products belong to the genus *Streptomyces* or related actinobacteria. Genetic engineering of these bacteria and the pathways that synthesize their valuable products often relies on serine integrases. To further improve the flexibility and efficiency of genome engineering via serine integrases, we explored whether multiple integrating vectors encoding orthogonally active serine integrases can be introduced simultaneously into *Streptomyces* recipients via conjugal transfer and integration. Pairwise combinations of *Escherichia coli* donors containing vectors encoding orthogonal serine integrases were used in each conjugation. Using donors containing plasmids (of various sizes) encoding either the φBT1 or the φC31 integration systems, we observed reproducible simultaneous plasmid integration into *Streptomyces coelicolor* and *Streptomyces lividans* at moderate frequencies after conjugation. This work demonstrated how site-specific recombination based on orthogonal serine integrases can save researchers time in genome engineering experiments in *Streptomyces *.

## Introduction

Phage-encoded serine integrases are a family of recombinases that mediate integration or excision of the phage genome into or out of the bacterial host chromosome [1]. Since the first serine integrases were described, such as those from *Streptomyces* phages φC31 and φBT1 [2, 3], and mycobacteriophage Bxb1 [4], these site-specific recombination systems have been used to develop genome integration vectors in bacteria and other organisms, including human and mammalian cell lines, plants and fungi [5–9]. Integration is the recombination between a specific sequence in an incoming circular DNA molecule (the *attP* site) and a specific site in the recipient genome (normally the endogenous *attB* site). Serine integrases bind to these sites, which are just 40–60 bp in length, and bring the sites together through protein–protein interactions. Within this complex the DNA sites are cut by integrase, maintaining the high-energy phosphate bond in the form of a phospho-serine link, and then reconfigure so that integrase can then rejoin the DNA strands but in a recombinant format. The end product is an integrated plasmid flanked by the recombinant sites, *attL* and *attR*. In some synthetic biology applications the excision reaction is also a useful feature [10–12]. Excision is the recombination, mediated by the serine integrase in the presence of a recombination directionality factor or RDF, of *attL* and *attR* to yield the reconstituted *attB* site and the *attP* site located on excised DNA. The mechanism of integration and excision by serine integrases has been described in detail in previous publications [8, 13, 14]. The efficiency, specificity and highly controllable nature of serine integrase-mediated recombination has led to these systems being widely applied in molecular genetics.

Although the mechanism of serine integrases has only been studied in a few members of this family, serine integrases can be detected in many phages and prophages. The pool of available *int/attP* sites is expanding, and the rate-limiting step to their use in heterologous systems has been the detection of the *attB* site, although this is made easier if the source of the *int/attP locus* is a prophage. The surge in synthetic biology applications of this protein family [15], such as in genetic memory devices [10–12], has resulted in a greatly increased number of characterized serine integrase systems.

*Streptomyces* spp. are known for their production of bioactive secondary metabolites or natural products, which are important in both healthcare and agriculture. Biosynthetic gene clusters (BGCs) of natural products are large and complex with multiple layers of regulation. Synthetic biology offers a way of exploring BGCs for which the product has not yet been characterized, that is, cloning the genes of the BGC into vectors that can be integrated into a well-studied heterologous *Streptomyces* host such as *Streptomyces coelicolor* or *Streptomyces lividans* and under the control of well-characterized promoters. These tractable hosts have worked well in the biosynthesis of complex natural products.

Strategies to manipulate BGCs *in vivo* have depended on the use of integration vectors derived from serine integrase *int/attP* loci. Haginaka *et al*. constructed two integrating plasmids, both containing the whole gene cluster of goadsporin and encoding either the φC31 *int*/*attP* locus or the TG1 *int*/*attP* locus. Introducing the two plasmids in consecutive conjugation experiments into the genome of the goadsporin producer, *Streptomyces* sp. TP-A0584, yielded a strain with two extra copies of the gene cluster. The recombinant strain was able tp produce 2.25-fold more goadsporin than the wild-type strain [16]. Li *et al*. applied a multiplexed site-specific genome engineering (MSGE) strategy to increase the production of pristinamycin II. In their work, two additional *attB* sites for φC31 and one additional *attB* site for φBT1 were introduced into the pristinamycin II producer, *Streptomyces pristinaespiralis*, using CRISPR/Cas9 technology. In consecutive conjugation experiments, additional copies of the pristinamycin II gene cluster were introduced into *S. pristinaespiralis* via plasmids encoding the entire gene cluster and either φC31 or φBT1 *int/attP* loci. In each conjugation, all the *attB* sites became efficiently occupied by the cognate integrating plasmids, resulting in a total of five copies of the BGC in the genome; two located in the innate *attB* sites and three in the additional *attB* sites. Notably, the production of pristinamycin II was elevated by four times in a 5 L bioreactor [17]. Elmore and colleagues designed a strategy called SAGE (Serine-integrase Assisted Genome Engineering), which enables iterative, site-specific integration of up to 10 different DNA constructs, into a poly-*attB* cassette that contains *attB* sequences for 10 serine integrases and has been inserted into host bacteria beforehand [18].

Previously our laboratory has used the erythromycin biosynthesis pathway as a model system to express BGCs in *Streptomyces* heterologous hosts [19]. The three polyketide synthase (PKS) genes *eryAI*, *eryAII* and *eryAIII* in the erythromycin BGC were cloned into three orthologous integrating plasmids, which were based on the *int*/*attP* loci from phages TG1, SV1 and φBT1, respectively. Following the integration, 6-deoxyerythronolide B (6-dEB), the first intermediate produced by the three PKS enzymes, could be detected in the fermentation broth. The results demonstrated that the sequential integration of multiple orthologous integrating vectors is a reliable method to clone large genes required to synthesize natural products.

In all these previous works (Table 1), researchers used different serine integrases and/or multiple integration loci (*attB* sites) to enhance hosts’ ability to accept more genes, but the integration processes were all carried out in an iterative format, that is, the integrating plasmids were introduced via consecutive conjugations. As different integrases only recombine their cognate recombination sites, their activities are expected to be entirely separate and independent of each other, that is, an integrase does not recognize or recombine the substrate sites of the other integrases. This orthogonality permits the use of different integrases in the same cell or *in vitro* recombination reaction, yielding entirely predictable recombinants, depending on the location of recombination sites. Moreover, the presence of orthogonal integration systems should not affect the efficiency of recombination of each one of those systems. The dynamics of conjugation to introduce pairs of different integrating plasmids into the same cell compartment, however, might affect efficiency.

**Table 1.** Previously published works using different serine integrases and/or multiple integration loci (*attB* sites) to engineer genomes iteratively

Reference	Hosts investigated	Integrases/*attB* sites employed
[16]	* Streptomyces* sp. TP-A0584	Native φC31 and TG1
[17] MSGE (multiplexed site-specific genome engineering)	*Streptomyces pristinaespiralis*	Native/additional φC31 and φBT1
[18] SAGE (serine-integrase assisted genome engineering)	* Pseudomonas fluorescens *, *Rhodopseudomonas palustris *	Cloned *attB* sites for Bxb1, φBT1, φC31, RV, TG1, R4, BL3, A118, MR11 and φ370
[19]	* Streptomyces coelicolor *, *Streptomyces lividans*	Native φBT1, SV1 and TG1

Here we tested a new strategy in which integrations could be multiplexed, thus introducing orthogonal integrating plasmids in a single conjugation. The results demonstrate that this strategy has the potential to be employed in synthetic biology and for natural product discovery, to engineer genomes more efficiently.

## Methods

### Bacterial strains and culture conditions

*Escherichia coli* strain Top10 (F-*mcrA* Δ(*mrr-hsdRMS-mcrBC*) φ80*lacZ*ΔM15 *ΔlacX74 nupG recA1 araD139* Δ(*ara-leu)7697 galE15 galK16 rpsL*(Str^R^) *endA1* λ^−^) was used for plasmid propagation and subcloning. *E. coli* strain ET12567(pUZ8002) was used as the donor host in plasmid conjugations from *E. coli* to *Streptomyces *. The *E. coli* strains were grown in Luria–Bertani broth (LB) or on LB agar at 37 °C.

*S. coelicolor* M1152 [*Δact Δred Δcpk Δcda rpoB*(*C1298T*)] [20] and *S. lividans* TK24 (*str-6* SLP2^−^ SLP3^−^) [21] were used as the recipients in conjugation. *Streptomyces* spore stocks were prepared from lawns grown on mannitol soya flour (MS) agar at 30^ ^°C, harvested in 20 % glycerol, and then frozen and stored at −38 °C. Prior to conjugation, *Streptomyces* spore stocks were diluted and spread on MS agar; after being incubated at 28 °C for 5 days, the number of colonies were counted to calculate spore concentrations (c.f.u. ml^−1^=number of colonies/volume plated×dilution factor). Counting was performed using plates with 25–250 colonies. Tryptic soy broth (TSB) medium was used for the preparation of genomic DNA [21]. The antibiotic concentrations for *E. coli* were 150 µg ml^−1^ hygromycin, 50 µg ml^−1^ apramycin, 50 µg ml^−1^ kanamycin and 100 µg ml^−1^ ampicillin, and for *Streptomyces* they were 50 µg ml^−1^ hygromycin, 50 µg ml^−1^ apramycin, 50 µg ml^−1^ kanamycin and 20 µg ml^−1^ erythromycin in DMSO for the selection of *S. coelicolor* M1152, 120 µg ml^−1^ erythromycin in DMSO for the selection of *S. lividans* TK24 and 25 µg ml^−1^ nalidixic acid for counterselection against *E. coli* on conjugation plates.

### DNA manipulation

*E. coli* transformation and gel electrophoresis were carried out as described previously [22]. Plasmid DNA extraction from *E. coli* was performed using a QIAprep spin miniprep kit (Qiagen, Germany) according to the manufacturer’s protocol. Restriction enzymes were purchased from New England BioLabs (NEB, USA) and were used according to the manufacturer’s instructions. In-Fusion cloning (Clontech, USA) and TA cloning (CloneJET PCR Cloning kit, Thermo Scientific, USA) were used for joining DNA fragments. Polymerase chain reaction (PCR) was carried out using Phusion High-Fidelity DNA Polymerase (NEB, USA) according to the manufacturer’s instructions. The plasmids used in this study are listed in Table 2 and the primers used are listed in Table 3.

**Table 2.** Plasmids used in this study

Plasmid	Description	Reference
pBF20	TG1 *int/attP, actIp-eryAI, tsr, ori/bla, aac(3)IV, oriT*	[19]
pBF22	SV1 *int/attP, actIp-eryAIII, tsr, ori/bla, aphII, oriT*	[19]
pBF24	φBT1 *int/attP, actIp-eryAII, tsr, ori/bla, ermE, oriT*	[19]
pHG4	TG1 *int/attP*, *bla/ori*, *aac(3)IV, oriT*	[23]
pHG5	SV1 *int/attP*, *bla/ori*, *aphII, oriT*	This study
pHG6	φBT1 *int/attP, bla/ori, ermE, oriT*	This study
pHG7	φC31 *int/attP, bla/ori, hygB, oriT*	This study
pHG2R2	φC31 *int/attP, actIp-eryBIV-eryBV, actIp-eryBVI, actIp-eryBIII-eryBII, actIp-eryBVII, tsr, ori/bla, actII-orf4/actIp-eryF, hygB, oriT*	[25]

**Table 3.** Oligonucleotides used in this study

Oligonucleotide	Sequence (5′−3′)
pHG5-for	CGAACGCATCGATTAATTAAGCGGCCGCCATATGTCTAGAGGTACCGAGCTCGCTAGCAGATCTATGAAACGAGACCTACCA
pHG5-rev	TGATTACGCCAAGCTTTCAGAAGAACTCGTCAAGAAGG
pHG6-for	CGAACGCATCGATTAATTAAGGTACCGAGCTCCATATGCCTCAGCGCATGCCATCAACCTCTGATTCCTCTCG
pHG6-rev	TGATTACGCCAAGCTTCGGGGCTTCAGACGTTTCG
pHG7-for	CGAACGCATCGATTAATTAAGCGGCCGCCATATGTCTAGAGGTACCGCTAGCGCATGCAGATCTCGGCCCGGGGCGTCAGGCG
pHG7-rev	TGATTACGCCAAGCTTCGCTACGCCGCTACGTC
pHG6-integration-for	AAGGGCAGCGATCAGCGC
pHG6-integration-Sc rev	CGACAGGGCGAGCCACAG
pHG6-integration-Sl rev	GTCGCCTATGACGTTCGGC
pHG7-integration-for	TCGAAGCCGTAAGGCGCC
pHG7-integration-Sc rev	GGCCTGCATCAGCTCGTCC
pHG7-integration-Sl rev	GGATGTCCTGGTAGCGCGG

### Construction of plasmids

**Fig. 1.**
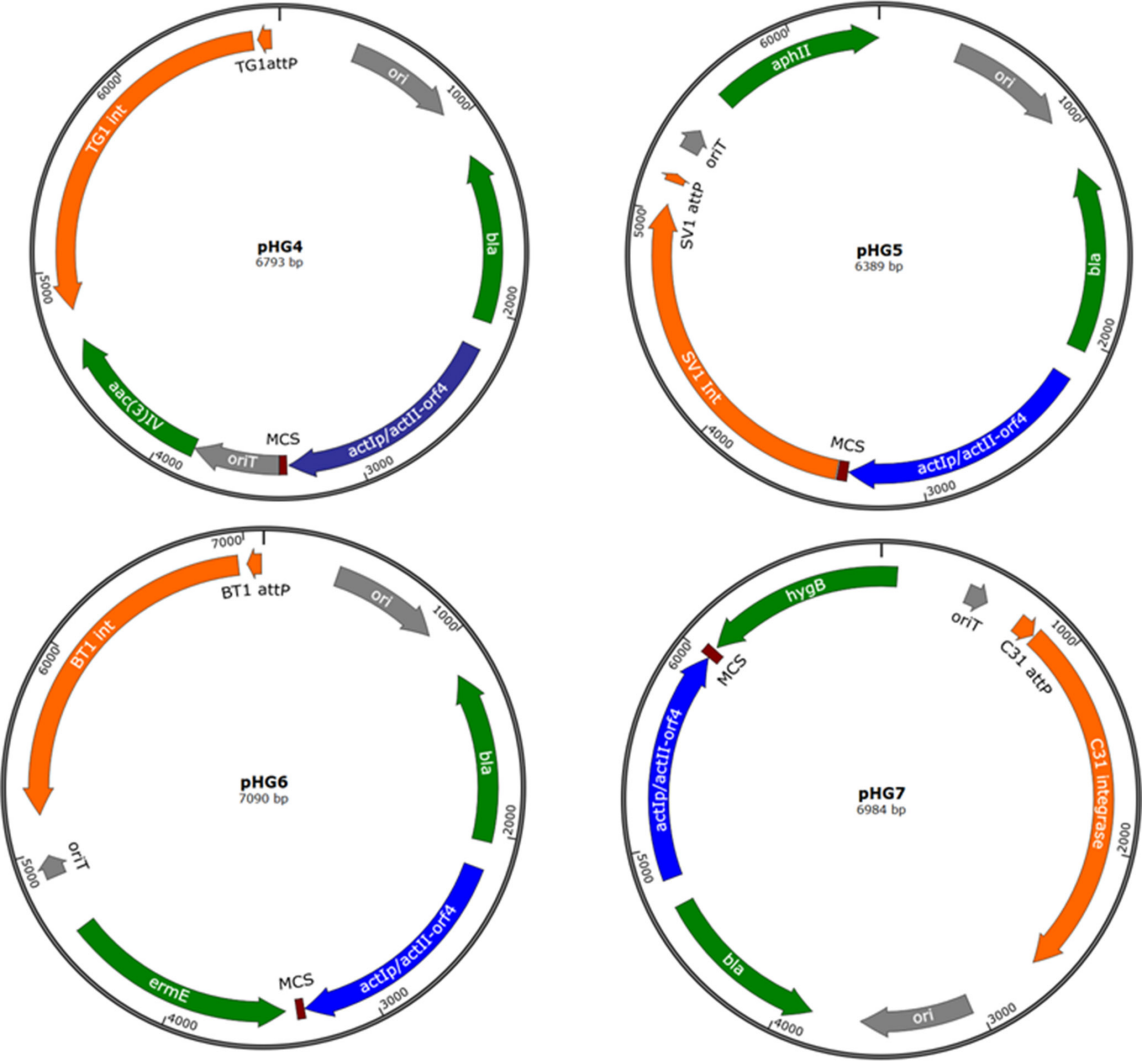
Plasmids used in this study.

Plasmids pHG4 [23], pHG5, pHG6 and pHG7 are all integrating vectors encoding the *int/attP* loci from TG1, SV1, φBT1 and φC31, respectively (Fig. 1). These four plasmids also encode compatible antibiotic resistance loci; apramycin resistance (*aac(3)IV*, pHG4), kanamycin resistance (*aphII*, pHG5), erythromycin resistance (*ermE*, pHG6) and hygromycin resistance (*hygB*, pHG7). Additionally, the plasmids all encode the expression cassette *actIp/actII-orf4* [24].

Plasmids pHG5, pHG6 and pHG7 were constructed as follows: the fragment containing SV1 *int*/*attP*, *oriT* and the kanamycin resistance gene was amplified from plasmid pBF22 [19] using the primer pair pHG5-for/pHG5-rev; the fragment containing erythromycin resistance gene, oriT and φBT1 *int*/*attP* was amplified from plasmid pBF24 [19] using the primer pair pHG6-for/pHG6-rev; and the fragment containing hygromycin resistance gene, oriT and φC31 *int*/*attP* was amplified from plasmid pBF27C [19] using the primer pair pHG7-for/pHG7-rev. Each PCR fragment was then inserted by In-Fusion cloning into pBF22 [19] cut with HindIII and PacI separately, to form the plasmids pHG5, pHG6 and pHG7 respectively.

Plasmids pBF20, pBF22, pBF24 [19] and pHG2R2 [25] were constructed in our previously published work.

### Intergeneric conjugation

*E. coli* ET12567 (pUZ8002) donors carrying desired plasmids were prepared as follows: a single colony was inoculated into 3 ml of LB broth with appropriate antibiotics for plasmid maintenance and incubated overnight at 37 °C and 200 r.p.m. The overnight cultures were diluted 100-fold into 10 ml of LB broth with appropriate antibiotics and incubated at 37 °C and 200 r.p.m. until an OD_600_ of 0.4 was reached. The cells were recovered by centrifugation (2700 ***g***, 10 min, 4 °C) and washed twice with chilled LB broth (10 ml) before resuspension in antibiotic-free LB broth (1 ml).

Germinating *Streptomyces* spores were used as plasmid recipients. Briefly, ~10^8^ spores were heat shocked in 2xYT medium (16 g l^−1^ tryptone, 10 g l^−1^ yeast extract and 5 g l^−1^ NaCl) at 50 °C for 10 min. Then prepared donor *E. coli* and recipient spores were mixed, plated onto MS agar plates containing 10 mM MgCl_2_ and incubated at 28 °C for 20 h. The MS plates were overlaid with water (1 ml) containing nalidixic acid (0.5 mg) plus the appropriate antibiotics for selection of exconjugants. Where required, each plate received: apramycin, 1.25 mg; kanamycin, 5 mg; erythromycin, 1.25 mg for *S. coelicolor* and 3.75 mg for *S. lividans*; and hygromycin, 1 mg. The overlaid plates were incubated at 28 °C until exconjugants could be counted. All conjugation experiments were performed in biological triplicate (*n*=3), and error bars denote standard deviation.

The resultant exconjugant colonies were replica plated onto MS agar containing nalidixic acid (25 µg ml^−1^) and appropriate selective antibiotics and incubated at 28 °C until sporulation. Spores collected from individual colonies were used directly as PCR templates. Conjugation efficiency was calculated as the number of exconjugants obtained from 10^8^ recipient cells.

## Results

### Optimization of the multiplexed conjugation

A drawback of using multiple expression plasmids is that consecutive rounds of conjugations are time-consuming, so we aimed to test whether it was possible to introduce two or more plasmids into *Streptomyces* hosts by multiplexing the conjugation donors in a single step. Plasmids pHG4, pHG5, pHG6 and pHG7 (Fig. 1) are approximately 6–7 kb and only contain essential vector elements. These basic integrating vectors were used to test the efficiency of multiplexed conjugations.

**Fig. 2.**
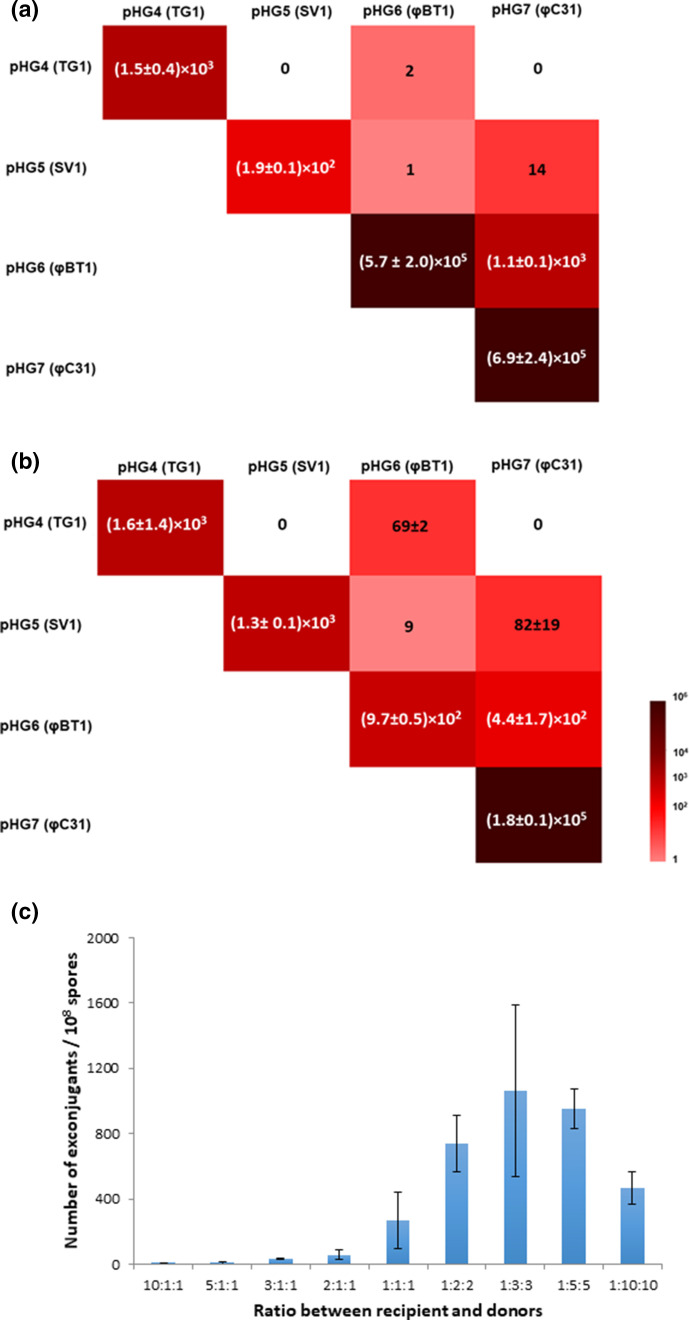
Conjugation frequencies of integrating plasmids in *Streptomyces* strains. The conjugation frequencies of pHG4, pHG5, pHG6 and pHG7 (in single and multiplexed conjugation) in (**a**) *S. coelicolor* M1152 and (**b**) *S. lividans* TK24. (**c**) Conjugation frequencies at different ratio of *S. coelicolor* spores and *E. coli* cells. The ratio value showed the ratio of *S. coelicolor* spores: *E. coli* ET12567 (pUZ8002) cells containing plasmid pHG6 : *E. coli* ET12567 (pUZ8002) cells containing plasmid pHG7. 1 : 1 : 1 means 10^8^ spores:10^8^ *E. coli* cells containing pHG6 : 10^8^ *E. coli* cells containing pHG7. The numbers in (**a**) and (**b**) indicated the numbers of exconjugants per 10^8^ spores.

First, the integrating plasmids were transferred into *S. coelicolor* individually to assay their conjugation and integration efficiencies. As *E. coli* donors containing either pHG6 (φBT1 *int/attP*) or pHG7 (φC31 *int/attP*) showed the highest efficiencies (Fig. 2a; diagonal cells), these donors were then selected to optimize the protocol for multiplexed conjugations.

For a multiplexed conjugation, the two *E. coli* donors containing either pHG6 or pHG7 were mixed together to simultaneously transfer plasmids into *S. coelicolor* M1152 in a single conjugation experiment. The effects of different ratios between *S. coelicolor* M1152 spores and *E. coli* donors on the transfer frequency were tested to find the optimal ratio (Fig. 2c). In the standard protocol, 10^8^ *E. coli* cells were conjugated with 10^8^ *S. coelicolor* M1152 spores. In this study, when a threefold excess of *E. coli* cells was used, the highest conjugation frequency could be achieved, and this ratio was used in all the following multiplexed mating attempts.

### Pairwise plasmid multiplex integration

Using the optimal ratio between donor cells and spores, *E. coli* donors containing the integrating plasmids pHG4, pHG5, pHG6 or pHG7 were tested pairwise in the multiplexed conjugation method with *S. coelicolor* M1152 (Fig. 2a) or *S. lividans* TK24 (Fig. 2b) as recipients. As expected, the number of exconjugants containing both integrating plasmids from the multiplexed donors in the conjugations was significantly reduced compared to using each donor individually. While use of the donor pairs containing either pHG6 and pHG7 led to reliable simultaneous transfer of both plasmids, *E. coli* pairs containing pHG4 (TG1 *int/attP, aac(3)IV*) or pHG5 (SV1 *int/attP, aphII*), and pHG4 or pHG7 (φC31 *int/attP, hyg*B) did not lead to any exconjugants that had received both plasmids.

**Fig. 3.**
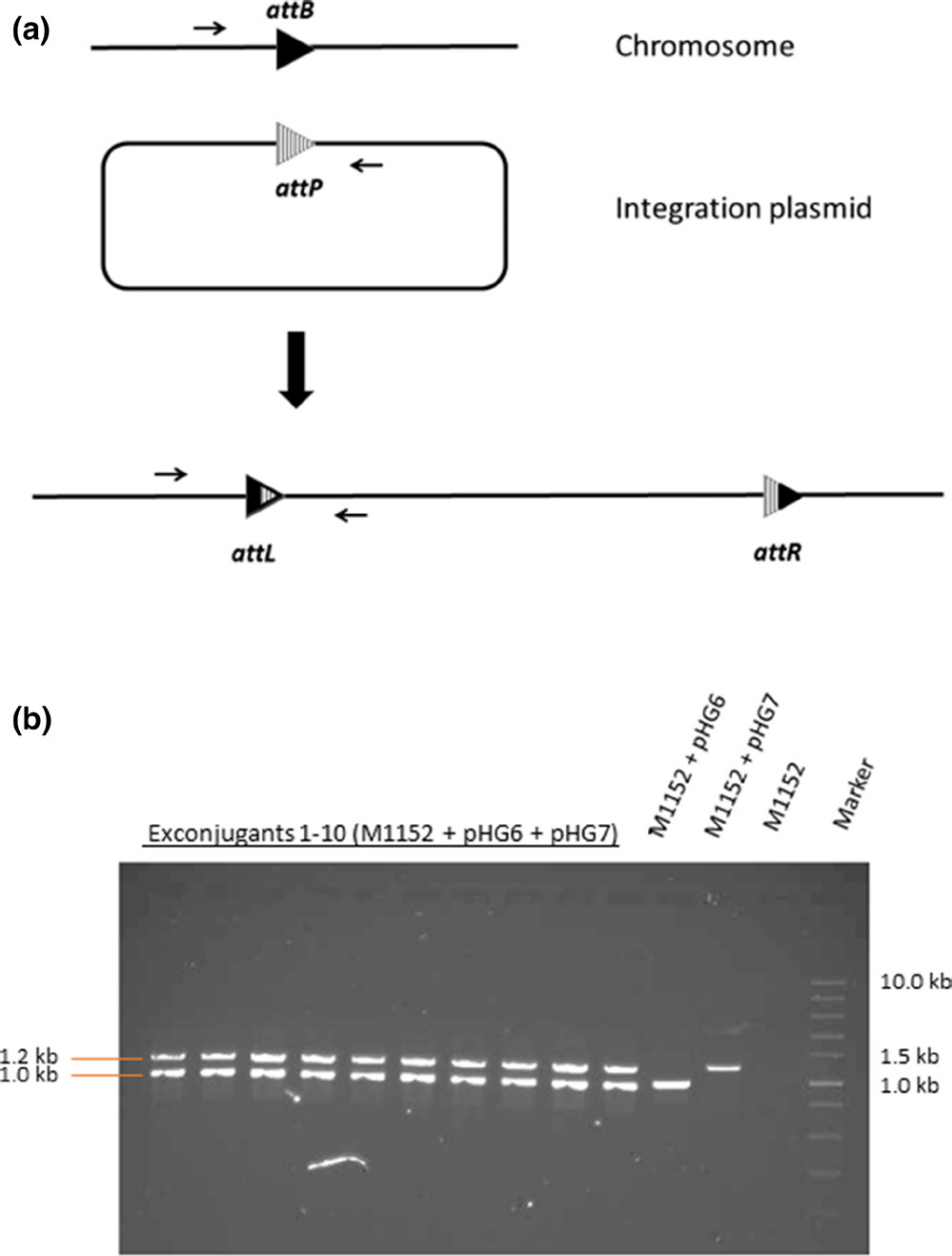
(**a**) The arrows show the primers designed to confirm the integration. (**b**) Two pairs of primers designed to confirm the integration of plasmids pHG6 and pHG7 were used to amplify fragments from exconjugants after multiplexed conjugation. Sizes of expected PCR products are 1.0 kb for pHG6 integration and 1.2 kb for pHG7 integration. Marker, NEB fast DNA ladder.

Overall the efficiencies of multiplexed conjugations using the donors in pairwise combinations were: pHG6 and pHG7 (φBT1 *int/attP* + φC31 *int/attP*) >pHG5 and pHG7 (SV1 *int/attP* + φC31 *int/attP*) >pHG4 and pHG6 (TG1 *int/attP* + φBT1 *int/attP*) >pHG5 and pHG6 (SV1 *int/attP* + φBT1 *int/attP*) in the *Streptomyces* strains tested. To confirm that correct site-specific integration had occurred in the exconjugants obtained after a multiplexed conjugation using *E. coli* donors containing pHG6 or pHG7, primers were designed to amplify the region across the φBT1 and φC31 *attL* sites after recombination (Fig. 3a). The exconjugants from the multiplexed conjugation were checked by colony PCR using the primer pairs pHG6-integration-for/pHG6-integration-Sc rev and pHG7-integration-for/pHG7-integration-Sc rev (for *S. coelicolor* M1152, Fig. 3) or pHG6-integration-for/pHG6-integration-Sl rev and pHG7-integration-for/pHG7-integration Sl rev (for *S. lividans* TK24, Fig. S1, available in the online version of this article). For *S. coelicolor* M1152-derived exconjugants, all of the 10 colonies picked randomly had pHG6 and pHG7 integrated correctly into the chromosome. For *S. lividans* TK24 exconjugants, the *attL* amplicons from both integrated pHG6 and pHG7 were obtained from seven out of eight colonies.

To gain insight into why the numbers of exconjugants containing both plasmids were so significantly reduced by multiplexed conjugations, we assayed the conjugation efficiency of each plasmid individually in the multiplexed experiments by selecting for just one of the plasmids being transferred. For individual plasmids, the efficiency of conjugation and integration was similar to when only one plasmid donor was used. This demonstrates that there is no interference between the donor cells.

The reliable ability to simultaneously transfer φBT1- and φC31-derived integrating vectors to *S. coelicolor* M1152 and *S. lividans* TK24 indicates that this is a practical method for future studies, saving a considerable amount of time.

### Multiplexed conjugations using larger plasmids containing biosynthetic genes

**Fig. 4.**
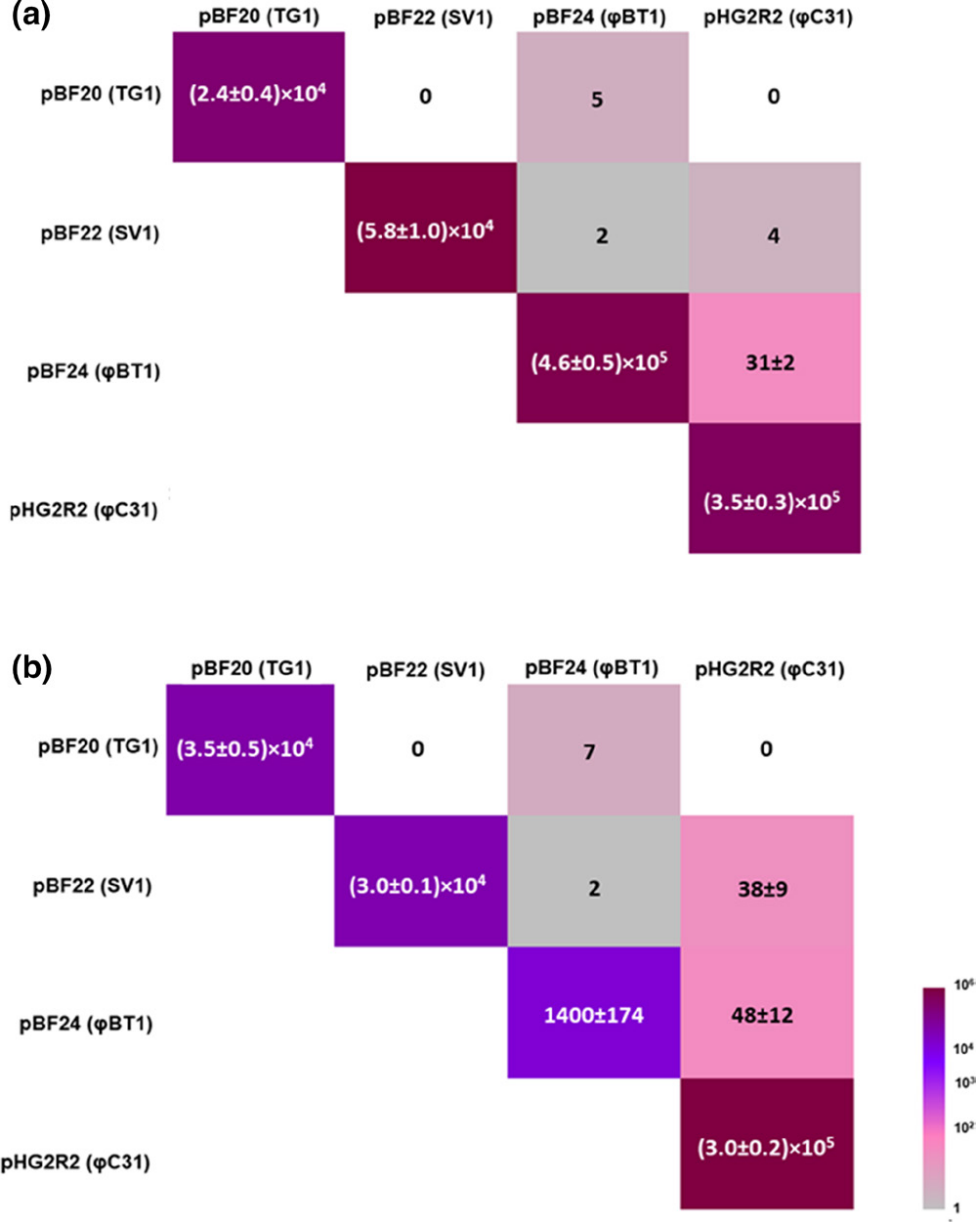
Conjugation frequencies of plasmids pBF20, pBF22, pBF24 and pHG2R2 (single and multiplexed conjugation) in (**a**) *S. coelicolor* M1152 and (**b**) *S. lividans* TK24. The numbers indicated the numbers of exconjugants per 10^8^ spores.

BGCs are usually highly complex, encoding many genes and sometimes contain very large, single multifunctional genes. Consequently BGCs are encoded by large DNA fragments. As the efficiency of DNA transformation by large plasmids can be reduced, multiplexed conjugation was tested with a plasmid set that encode large biosynthetic genes, i.e. plasmids pBF20, pBF22, pBF24 [19] and pHG2R2 [25]. The size of these four plasmids is between 17 and 19 kb and they encode biosynthetic genes for erythromycin biosynthesis. With these much larger plasmids, the number of exconjugants that had received the two plasmids was much lower than the experiments using just the empty vectors. The most efficient combination, pBF24 and pHG2R2 (φBT1 *int/attP* + φC31 *int/attP*), however still showed that simultaneous transfer of two plasmids is feasible, even though the plasmids are nearly 20 kb (Fig. 4).

As before, the sites of integration for the plasmids after multiplexed conjugation were verified by colony PCR using the primer pairs pHG6-integration-for/pHG6-integration-Sc rev and pHG7-integration-for/pHG7-integration Sc rev for *S. coelicolor* M1152 (Fig. S2a), and the primer pairs pHG6-integration-for/pHG6-integration-Sl rev and pHG7-integration-for/pHG7-integration-Sl rev for *S. lividans* TK24 (Fig. S2b). The *attL*s for both plasmids could be amplified from the majority of exconjugants, showing the efficiency of the simultaneous integration, even with large inserts, from a single mating experiment, indicating that this is still a practical method.

## Discussion

In this study, we tested the feasibility of simultaneous conjugation and integration of plasmids into *Streptomyces* sp. Conjugation is one of the most commonly used methods of bacterial gene transfer and in *Streptomyces.* It is widely used to deliver integrating plasmids that depend on the presence of a phage-derived *int/attP* locus. Although the simultaneous transfer and integration of two plasmids into *Streptomyces* in a single conjugation experiment is substantially less efficient than the transfer of each plasmid in standard bi-parental matings, the observed numbers of exconjugants obtained make a multiplexed conjugation step a viable proposition in genetic engineering methodology. Integrating plasmids based on φBT1 and φC31 *int/attP* loci showed the highest efficiencies of transfer in a multiplexed conjugation. The plasmids based on these *int/attP* sites would even allow the simultaneous conjugation and integration of very large plasmids. In previous work requiring the conjugation and integration of multiple plasmids with orthologous *int/attP* loci, each plasmid was introduced in series using repeated rounds of conjugative transfer. Our results showed that when the plasmids being delivered are derived from φBT1 and φC31 *int/attP* loci, only a single conjugation using two *E. coli* donors is required, obviating the need for separate rounds of conjugation.

While we were preparing this paper, Ko and colleagues published their findings [26]. They constructed vectors encoding orthogonal resistances, integrases (and containing their cognate *attP* sites) and origins of replications and introduced these into a single *E. coli* donor that in a conjugation step could then be introduced into various *Streptomyces* hosts. In our study, the integrating vectors used contained the same origin of replication and the plasmids were introduced from combinations of *E. coli* donors. Both studies used a single conjugation step to deliver integrating plasmids.

Ko’s work and our study achieved similar results. As described above, conjugations in which plasmids containing the φBT1 and φC31 *int/attP* loci are simultaneously transferred yield the highest numbers of exconjugants. We did not test the φOZJ integration system, since it was characterized for the first time by Ko *et al*. However, their outcomes suggest that φOZJ might also work in our multiplexed conjugation and integration system.

We attempted multiplexed conjugations that sought to simultaneously transfer three plasmids into *Streptomyces* hosts. The combinations tested contained the *int/attP* loci from φBT1 and φC31, and *int/attP* from either TG1 or SV1. No exconjugants were obtained with either combination using the same procedure described for the conjugations using pairs of donors. Increasing the concentration of the donors by 10× to be in a standard bi-parental conjugation protocol also failed to produce exconjugants containing all three plasmids (data not shown). Similarly, Ko’s tetra-parental (three *E. coli* donors containing plasmids encoding the integration loci from φOZJ, φBT1 and φC31, into a *Streptomyces* host) mating attempt failed to yield exconjugants, but if the three plasmids to be transferred are all present in the same *E. coli* donor (via compatible origins of replication and orthogonal resistance markers), exconjugants containing all three plasmids are obtained. Based on these results, Ko *et al*. suggested that during conjugation multiple plasmids can be transferred via the conjugation apparatus formed in a donor–recipient interaction. Our data indicate that multiple independent conjugation events also occur, but even though the number of exconjugants may not be as high as when using a single donor, there may be advantages to using the same high copy replication origin for plasmid construction and simply mixing the two donors.

In summary, we optimized the multiplexed conjugation and integration method, which can simultaneously introduce two plasmids encoding orthogonal integrating loci into *Streptomyces *. We also demonstrated that the method is robust even when introducing large plasmids, a likely scenario when engineering biosynthetic gene clusters.

## Supplementary Data

Supplementary material 1

## References

[1] Rutherford K, Van Duyne GD (2014). The ins and outs of serine integrase site-specific recombination. Curr Opin Struct Biol.

[2] Thorpe HM, Smith MC (1998). *In vitro* site-specific integration of bacteriophage DNA catalyzed by a recombinase of the resolvase/invertase family. Proc Natl Acad Sci U S A.

[3] Gregory MA, Till R, Smith MCM (2003). Integration site for *Streptomyces phage* phiBT1 and development of site-specific integrating vectors. J Bacteriol.

[4] Kim AI, Ghosh P, Aaron MA, Bibb LA, Jain S (2003). *Mycobacteriophage* Bxb1 integrates into the *Mycobacterium smegmatis* groEL1 gene. Mol Microbiol.

[5] Hong Y, Hondalus MK (2008). Site-specific integration of *Streptomyces* PhiC31 integrase-based vectors in the chromosome of *Rhodococcus equi*. FEMS Microbiol Lett.

[6] Saha S, Zhang W, Zhang G, Zhu Y, Chen Y (2017). Activation and characterization of a cryptic gene cluster reveals a cyclization cascade for polycyclic tetramate macrolactams. Chem Sci.

[7] Sosio M, Giusino F, Cappellano C, Bossi E, Puglia AM (2000). Artificial chromosomes for antibiotic-producing actinomycetes. Nat Biotechnol.

[8] Fogg PCM, Colloms S, Rosser S, Stark M, Smith MCM (2014). New applications for phage integrases. J Mol Biol.

[9] Xu Z, Thomas L, Davies B, Chalmers R, Smith M (2013). Accuracy and efficiency define Bxb1 integrase as the best of fifteen candidate serine recombinases for the integration of DNA into the human genome. BMC Biotechnol.

[10] Gomide MS, Sales TT, Barros LRC, Limia CG, de Oliveira MA (2020). Genetic switches designed for eukaryotic cells and controlled by serine integrases. Commun Biol.

[11] Zhao J, Pokhilko A, Ebenhöh O, Rosser SJ, Colloms SD (2019). A single-input binary counting module based on serine integrase site-specific recombination. Nucleic Acids Res.

[12] Yang L, Nielsen AAK, Fernandez-Rodriguez J, McClune CJ, Laub MT (2014). Permanent genetic memory with >1-byte capacity. Nat Methods.

[13] Colloms SD, Merrick CA, Olorunniji FJ, Stark WM, Smith MCM (2014). Rapid metabolic pathway assembly and modification using serine integrase site-specific recombination. Nucleic Acids Res.

[14] Smith MC (2015). Phage‐encoded serine integrases and other large serine recombinases. Mobile DNA III.

[15] Stark WM (2017). Making serine integrases work for us. Curr Opin Microbiol.

[16] Haginaka K, Asamizu S, Ozaki T, Igarashi Y, Furumai T (2014). Genetic approaches to generate hyper-producing strains of goadsporin: the relationships between productivity and gene duplication in secondary metabolite biosynthesis. Biosci Biotechnol Biochem.

[17] Li L, Zheng G, Chen J, Ge M, Jiang W (2017). Multiplexed site-specific genome engineering for overproducing bioactive secondary metabolites in actinomycetes. Metab Eng.

[18] Elmore JR, Dexter GN, Francis R, Riley L, Huenemann J (2020). The SAGE genetic toolkit enables highly efficient, iterative site-specific genome engineering in bacteria. bioRxiv.

[19] Fayed B, Ashford DA, Hashem AM, Amin MA, El Gazayerly ON (2015). Multiplexed integrating plasmids for engineering of the erythromycin gene cluster for expression in *Streptomyces* spp. and combinatorial biosynthesis. Appl Environ Microbiol.

[20] Gomez-Escribano JP, Bibb MJ (2011). Engineering *Streptomyces coelicolor* for heterologous expression of secondary metabolite gene clusters. Microb Biotechnol.

[21] Kieser T, Bibb MJ, Buttner MJ, Chater KF, Hopwood DA (2000). Practical Streptomyces Genetics.

[22] Sambrook J, Fritsch EF, Maniatis T (1989). Molecular cloning: a laboratory manual. Cold spring harbor laboratory press.

[23] Gao H, Murugesan B, Hoßbach J, Evans SK, Stark WM (2019). Integrating vectors for genetic studies in the rare Actinomycete *Amycolatopsis marina*. BMC Biotechnol.

[24] Rowe CJ, Cortés J, Gaisser S, Staunton J, Leadlay PF (1998). Construction of new vectors for high-level expression in actinomycetes. Gene.

[25] Gao H, Taylor G, Evans SK, Fogg PCM, Smith MCM (2020). Application of serine integrases for secondary metabolite pathway assembly in Streptomyces. Synth Syst Biotechnol.

[26] Ko B, D’Alessandro J, Douangkeomany L, Stumpf S, deButts A (2020). Construction of a new integrating vector from actinophage ϕOZJ and its use in multiplex *Streptomyces* transformation. J Ind Microbiol Biotechnol.

