# Supplementary material for: Use of orthogonal serine integrases to multiplex plasmid conjugation and integration from E. coli into Streptomyces
Source: Access Microbiol. 2021 Dec 8;3(12):000291. doi: 10.1099/acmi.0.000291 (PMC8749152; doi:10.1099/acmi.0.000291)
Supplement: Supplementary material 1 [file acmi-3-0291-s001.pdf]

Use of Orthogonal Serine Integrases to Multiplex Plasmid Conjugation and Integration from *E. coli* into *Streptomyces*

Hong Gao[1,2,3,*], Margaret C. M. Smith[1,*]

1. Department of Biology, University of York, York, United Kingdom, YO10 5DD
2. School of Health and Life Sciences, Teesside University, Middlesbrough, UK, TS1 3BA
3. National Horizons Centre, Teesside University, Darlington, UK, DL1 1HG

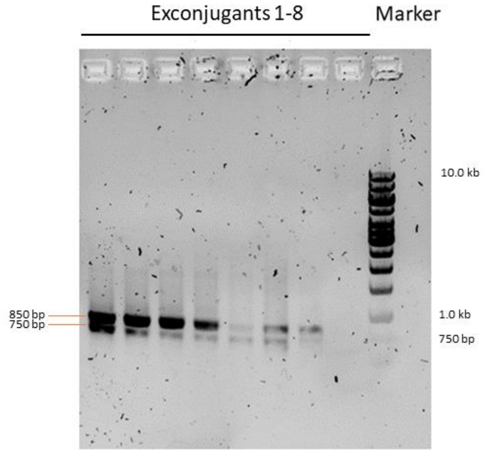

Figure S1. PCR reactions to confirm the multiplexed integration of plasmids pHG6 and pHG7 in *S. lividans* TK24. Sizes of expected PCR products are 750 bp for pHG6 integration and 850 bp for pHG7 integration. Marker: GeneRuler 1 kb DNA Ladder.

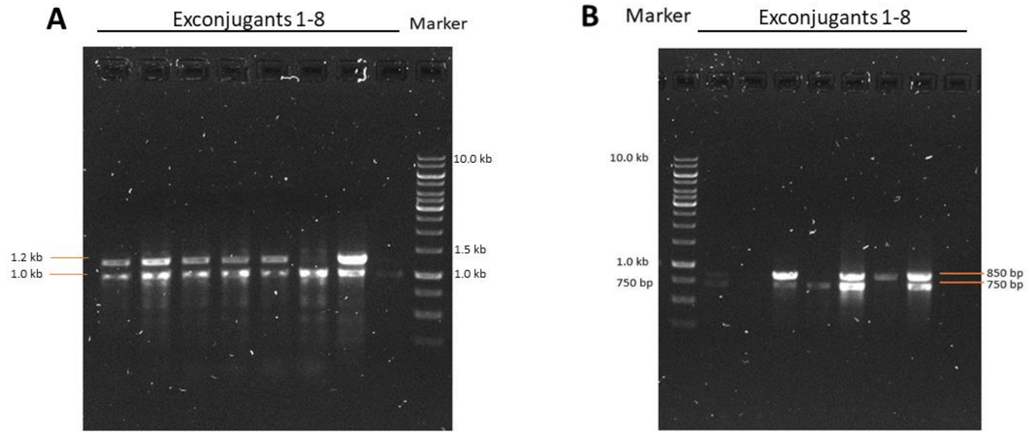

Figure S2. PCR reactions to confirm the multiplexed integration of plasmids pBF24 and pHG2R2 in (A) *S. coelicolor* M1152 (Sizes of expected PCR products are 1.0 kb for pHG6 integration and 1.2 kb for pHG7 integration), and (B) *S. lividans* TK24 (Sizes of expected PCR products are 750 bp for pHG6 integration and 850 bp for pHG7 integration). Marker: GeneRuler 1 kb DNA Ladder.
